# Inactivation and reactivation of ribonuclease A studied by computer simulation

**DOI:** 10.1098/rsob.120088

**Published:** 2012-07

**Authors:** Gavin M. Seddon, Robert P. Bywater

**Affiliations:** 1 Adelard Institute, Manchester M29 7FZ, UK; 2 Magdalen College, Oxford OX1 4AU, UK

**Keywords:** protein structure, protein folding, molecular dynamics, enzyme deactivation, enzyme reactivation

## Abstract

The year 2011 marked the half-centenary of the publication of what came to be known as the Anfinsen postulate, that the tertiary structure of a folded protein is prescribed fully by the sequence of its constituent amino acid residues. This postulate has become established as a *credo*, and, indeed, no contradictions seem to have been found to date. However, the experiments that led to this postulate were conducted on only a single protein, bovine ribonuclease A (RNAse). We conduct molecular dynamics (MD) simulations on this protein with the aim of mimicking this experiment as well as making the methodology available for use with basically any protein. There have been many attempts to model denaturation and refolding processes of globular proteins *in silico* using MD, but only a few examples where disulphide-bond containing proteins were studied. We took the view that if the reductive deactivation and oxidative reactivation processes of RNAse could be modelled *in silico,* this would provide valuable insights into the workings of the classical Anfinsen experiment.

## 2. Introduction

In 1961, Anfinsen *et al*. [1] reported the full recovery of activity in an enzyme, bovine ribonuclease A (RNAse), after urea/reductive deactivation. The recovery of activity was accomplished by removing the urea by dialysis and allowing the enzyme solution to re-oxidize slowly in air. In this carefully conducted study, there were no additives present that might inadvertently have promoted the correct folding. One of the intriguing features of this experiment was that it required that the protein explore configuration space thoroughly enough so that the correct disulphide connectivity (four correctly formed SS-bonds out of 28 possible) could be arrived at. The result was so striking that it led to the proposal that the information for correct formation of the disulphide bridges, and of the protein secondary and tertiary structure was contained in the sequence itself. In the half-century since the annunciation of the Anfinsen postulate, there has appeared no evidence which contradicts it, but neither, seemingly, has there been any systematic experimental work on other proteins which would have further established its validity.

There have been many attempts [2–5] to model the unfolding and refolding processes of globular proteins using molecular dynamics (MD). However, these have mostly concerned very small proteins with molecular weight (MW) approximately 4 and 6.5 [5], 7.6 [2] and 7.5 kDa [3], respectively, with one example of a somewhat larger protein with MW 18.6 kDa [4]. In a companion paper to this one [6], we study a large and complex protein with MW approximately 34 kDa, which is far larger than has been accomplished by any other group. Here we use MD to study RNAse, which is smaller, MW 13.7 kDa, but with the added complication that it contains disulphide bridges. Proteins with disulphide bridges have not widely been studied before by MD, but we wished to reproduce the Anfinsen experiment *in silico*. Accordingly, we study RNAse in both the reduced and oxidized forms and use molecular modelling to carry out the breakage and re-formation of the disulphide bridges as explained below.

Disulphide bonds have an important role to play in several different enzyme functions including the maintenance of tertiary structure and catalytic activity. In RNAse, the key catalytic events are catered for by a pair of histidine residues (His 12 and His 119). Therefore, we track Cys–Cys and His–His distances throughout the unfolding and recovery steps.

## 3. Results

Key structural parameters for significant species along the inactivation and reactivation pathways are summarized in tables 1 and 2. When RNAse is reduced, there is at first no detectable change in secondary structures. There is some readjustment among the SG–SG distances with even one case of a pair coming into closer proximity, but what this suggests is that the integrity of the three-dimensional structure of the enzyme is not maintained by the disulphide bridges, which is also in total agreement with the Anfinsen experiment.

**Table 1.** Secondary structures and cavity volumes of key structures on the inactivation and reactivation pathways.

structure	helix	strand	turn	coil	3_10
1kf3orig^a^	17.70	33.07	25.00	20.97	3.23
1kf3sh^b^	17.70	33.07	25.00	20.97	3.23
1k3suw0^c^	14.52	16.13	26.61	42.74	0
1k3suw5^d^	15.30	19.36	27.42	37.90	0
1kf3rec9.5^e^	20.16	33.87	23.39	18.55	4.03
1k3anfo^f^	20.16	28.23	17.74	29.03	4.03

^a^The starting structure, essentially 1kf3 with crystal waters and sulphate removed.

^b^The starting structure with all four disulphides ruptured by reduction.

^c^The reduced structure immersed in urea/water at 0 ps.

^d^The structure after 5 ps in urea/water.

^e^The structure after 9 ns in water.

^f^The 9 ns structure after quality-checking and correction.

**Table 2.** SG–SG distances in Cys residues and His 12 ND1 and His 119 NE2 of key structures on the inactivation and reactivation pathways.

1kf3orig^a^		
26 C	84 C	2.03
40 C	95 C	2.01
58 C	110 C	2.01
65 C	72 C	2.02
12 H	119 H	6.37
1kf3sh^b^		
26 C	84 C	2.09
40 C	95 C	2.16
58 C	110 C	2.20
65 C	72 C	1.53
12 H	119 H	6.40
1kf3suw0^c^		
26 C	84 C	4.22
40 C	95 C	4.03
58 C	110 C	8.01
65 C	72 C	4.15
12 H	119 H	8.14
1k3suw5^d^		
26 C	84 C	3.92
40 C	95 C	6.86
58 C	110 C	6.68
65 C	72 C	3.63
12 H	119 H	9.30
1kf3rec9.5^e^		
26 C	84 C	6.10
40 C	95 C	21.21
58 C	110 C	3.43
65 C	72 C	3.66
12 H	119 H	2.88
1k3anfo^f^		
26 C	84 C	2.08
40 C	95 C	2.09
58 C	110 C	2.08
65 C	72 C	2.07
12 H	119 H	3.40

^a^The starting structure, essentially 1kf3 with crystal waters and sulphate removed.

^b^The starting structure with all four disulphides ruptured by reduction.

^c^The reduced structure immersed in urea/water at 0 ps.

^d^The structure after 5 ps in urea/water.

^e^The structure after 9 ns in water.

^f^The 9 ns structure after quality-checking and correction.

However, when immersed in a urea solution, there is an immediate relaxation of the structure. All SG–SG distances expand as does the His 12 ND1–His 119 NE2 distance, which is diagnostic of enzyme activity [7] in ribonuclease. It would be easy to make the assumption that this simply leads to a continued process of unfolding with concomitant expansion of the three-dimensional structure and of the distances mentioned above, but, in fact, nothing could be further from the truth. When the protein structure is allowed to evolve for extended periods up to 5 ps in urea, and then transferred to water for 9 ns, there is, instead of unfolding, a return towards the native structure, and even a bit beyond, in the case of an α-helix and strand (table 1).

As the trajectory proceeds to 5 ps in urea/water, the all-important His 12 ND1–His 119 NE2 spacing widens; while some SG–SG distances expand, others contract (data in table 2). When extended further to 9 ns in water, there is a dramatic contraction in the His 12 ND1–His 119 NE2 spacing to a displacement even shorter than in the native structure. Three of the SG–SG distances contract while the fourth expands dramatically. The small amount of 3_10 helix disappears and later reappears, more or less in synchrony with the way that the α-helix behaves.

**Figure 1.**
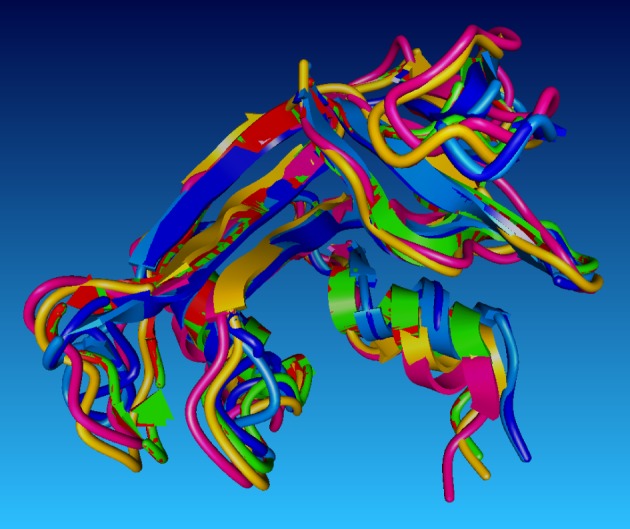
Superposed structures for ribonuclease at various stages of the simulation. The key to the names used here is to be found in the footnote of the table: blue, 1kf3orig; red, 1kf3sh; green, 1kf3suw5; purple, 1kf3anfo.

Finally, we provide r.m.s. data for all pairwise superposed structures in table 3, from which it can be seen that after inactivation followed by the reactivation procedure, the final structure approaches the initial structure. Four of the five superposed structures are shown in figure 1.

**Table 3.** Pairwise root mean square differences between the superposed structures.

r.m.s. Å	1kf3orig	1kf3sh	1k3suw0	1k3suw5	1kf3rec9.5	1k3anfo
1kf3orig^a^	0	0.10	1.67	2.48	4.11	2.82
1kf3sh^b^		0	1.81	2.63	4.34	3.14
1k3suw0^c^			0	1.83	4.53	3.41
1k3suw5^d^				0	4.79	3.72
1kf3rec9.5^e^					0	3.38
1k3anfo^f^						0

^a^The starting structure, essentially 1kf3 with crystal waters and sulphate removed.

^b^The starting structure with all four disulphides ruptured by reduction.

^c^The reduced structure immersed in urea/water at 0 ps.

^d^The structure after 5 ps in urea/water.

^e^Recovered structure after 9 ns in water.

^f^The 9 ns structure after quality-checking and correction.

## 4. Conclusions

We have conducted a series of simulations as described above and consider the final state we arrive at (1kf3anfo) to represent the reactivated state of the enzyme. Our claim rests upon the very similar secondary structures that are obtained plus the fact that the disulphide bonds have re-formed with the correct connectivity. The critical His 12 ND1–His 119 NE2 distance is somewhat smaller than the original value. We have recovered a tightly packed structure (a critical requirement of properly folded protein structures that is often overlooked by protein folders). However, we do not expect the final structure to converge exactly upon the initial structure. The latter is a crystal structure that typically contains packing defects [8] and it is in fact impossible to reconstruct that structure without knowing the crystalline space group and arrangement of molecules in the lattice. Further, there may be some ambiguity as to which structure one obtains finally, upon refolding, since there is usually not a single ‘native’ state, but rather two (or more) states accessible to the protein, reflecting the way that they can switch between, for example, active or inactive conformations [9]. We have of course not covered the entire folding landscape for the protein, but at least an important part of it, from deactivation back to a structure with all the attributes of a fully active structure. Anfinsen was mainly concerned with the loss of activity and its recovery; nevertheless, he conducted experiments which convincingly showed that there was a considerable degree of unfolding [10], quite possibly beyond the stage that we reached, but we have no way of knowing this exactly. Our main concern here is with the deactivation and subsequent reactivation of the enzyme, as detected in our case by the critical His 12 and His 119 distance. We avoid the use of the term denaturation, which could be extended to much more drastic and prolonged treatment with denaturants leading to structures from which recovery may be impossible. Thus, while the Anfinsen postulate holds for the incipient denaturation that we are studying, and probably some way beyond that, it may not hold for all unfolded or misfolded structures.^1^

The postulate that the final three-dimensional conformation of globular proteins is prescribed by the amino acid sequence has not been contradicted throughout some 50 years of protein folding studies and our results are in accordance with that finding. The Anfinsen experiment was conducted for only a single protein. Since our method is based on an *in silico* approach, it does not require a great deal of preparation in order to conduct similar studies on different enzymes, which we have already embarked on.

## 5. Methods

### 5.1. Molecular dynamics simulations

All MD simulations were conducted under the Amber 03 force field [11] as implemented in the protein modelling package Yasara [12]. A single copy of the RNAse structure PDB I.d. 1 kf3 was enclosed in a rectangular box of dimensions 48.5 × 54.0 × 43.6 Å. This was filled with copies of a single urea molecule. The number of urea molecules generated by Yasara to just fill the box was 1058. This file was saved and then edited to remove all but 159 urea molecules; this number was calculated to represent a urea concentration of 8 M when the remaining 899 molecules were replaced by water (6334 water molecules). The 159 urea molecules were chosen so as to be spread out as evenly as possible in the box. The selection procedure involved using the Linux sort command to sort the urea molecule entries along the three coordinate axes of the box and then eliminating entries that clustered too closely, first along X, then Y and finally Z, in such a manner as to ensure a well-spaced-out distribution of the urea molecules in the box, avoiding any clustering. Yasara was then used once more to fill the empty regions with water. The final density in the box, which contained protein, urea and water, was 1.6, corresponding to a urea concentration of 8 M.

To conduct the simulations on reduced ribonuclease, the untreated enzyme molecule was extracted from the box and replaced with the reduced form produced as described in the next section. The pressure was set at 1013 Hpa and the box was minimized with Amber 03 at 25°C for 1 ps and run under MD at 60°C, saving coordinates every 100 ps and stopping at 9 ns. Movies of both these steps are available for inspection on www.adelard.org.uk/movies.

Simulations of the recovery of activity required urea to be removed and replaced with water. The treated solution was minimized/equilibrated to ensure that the change in environment did not affect the system adversely.

### 5.2. Reduction of disulphide bridges

This was carried out using the protein modelling program What if [13], which has a choice of routines whereby the program considers all cysteine residues to be reduced or alternatively oxidized as in a disulphide bridge, the connectivity in the latter case being dictated according to the mutual proximity of the respective SG atoms. The latter is the default procedure; the former is an added feature intended to ensure that a disulphide bridge is not automatically assumed merely on the basis of this proximity.

## 7. Endnote

1 Indeed, nature has developed a specific strategy for recovering from such mishaps, the so-called chaperones.
